# A Wheat β-Patchoulene Synthase Confers Resistance against Herbivory in Transgenic Arabidopsis

**DOI:** 10.3390/genes10060441

**Published:** 2019-06-10

**Authors:** Qingyu Pu, Jin Liang, Qinqin Shen, Jingye Fu, Zhien Pu, Jiang Liu, Xuegui Wang, Qiang Wang

**Affiliations:** Institute of Ecological Agriculture, College of Agronomy, Sichuan Agricultural University, Chengdu 611130, China; cnpuqingyu@sina.com (Q.P.); L_Jinch@126.com (J.L.); shenqin2000@sina.cn (Q.S.); fjy0204@sina.com (J.F.); puzhien@sicau.edu.cn (Z.P.); jiangliu@sicau.edu.cn (J.L.); wangxuegui@sicau.edu.cn (X.W.)

**Keywords:** arabidopsis, herbivory, patchoulene, resistance, sesquiterpene, wheat

## Abstract

Terpenoids play important roles in plant defense. Although some terpene synthases have been characterized, terpenoids and their biosynthesis in wheat (*Triticum aestivum* L.) still remain largely unknown. Here, we describe the identification of a terpene synthase gene in wheat. It encodes a sesquiterpene synthase that catalyzes β-patchoulene formation with *E,E*-farnesyl diphosphate (FPP) as the substrate, thus named as TaPS. TaPS exhibits inducible expression in wheat in response to various elicitations. Particularly, alamethicin treatment strongly induces *TaPS* gene expression and β-patchoulene accumulation in wheat. Overexpression of *TaPS* in Arabidopsis successfully produces β-patchoulene, verifying the biochemical function of TaPS in planta. Furthermore, these transgenic Arabidopsis plants exhibit resistance against herbivory by repelling beet armyworm larvae feeding, thereby indicating anti-herbivory activity of β-patchoulene. The catalytic mechanism of TaPS is also explored by homology modeling and site-directed mutagenesis. Two key amino acids are identified to act in protonation and stability of intermediates and product formation. Taken together, one wheat sesquiterpene synthase is identified as β-patchoulene synthase. TaPS exhibits inducible gene expression and the sesquiterpene β-patchoulene is involved in repelling insect infestation.

## 1. Introduction

Plants are sessile organisms and cannot escape adverse environmental conditions. Diverse defense mechanisms have evolved in plants in response to differential stresses. Chemical defense is a pivotal mechanism and involves a large number of specialized metabolites [1]. Chemical defense protects plants directly or indirectly [2]. The direct chemical defense involves metabolites with toxic or growth inhibitory effects on pests or pathogens, like phytoalexins impairing pathogen spore germination and/or growth, as well as toxic or repellent metabolites against insects [3]. Furthermore, indirect chemical defense recruits volatiles to attract parasitoids or predators of attacking herbivores [4].

Terpenes/terpenoids are most abundant natural products and have been identified to play important roles in plant chemical defense. A number of terpenoid phytoalexins exhibit anti-microbial activities in plants, such as momilactones, phytocassanes and oryzalexins in rice [5,6], zealexins, kauralexins and dolabralexins in maize [7,8,9,10], capsidiol in tobacco [11]. Other terpene olefins were also involved in resistance against pathogens, like limonene, β-caryophyllene [12,13,14]. In chemical defense against herbivory, volatile terpenes play the major role to repel insects directly or attract predators of feeding pests. β-farnesene, β-caryophyllene, two homoterpenes (*E*)-3,8-dimethyl-1,4,7-nonatriene (DMNT) and (*E,E*)-4,8,12-trimethyltrideca-1,3,7,11-tetraene (TMTT) have been mainly studied for their anti-herbivory effects [15,16,17,18,19]. In addition, some terpenoid phytoalexins also exhibited antifeedant activities against pests [9].

Terpenoid biosynthesis has been explored under various aspects [20]. In plant cells, terpenoid precursors isopentenyl pyrophosphate (IPP) and dimethylallyl pyrophosphate (DMAPP) are synthesized via the plastid-localized methylerythritol 4-phosphate (MEP) pathway or cytosolic mevalonic acid (MVA) pathway (Figure S1) [21]. The MEP pathway utilizes pyruvic acid and 3-phosphoglyceraldehyde to produce IPP and DMAPP in plastids to yield geranyl diphosphate (GPP), the precursor of monoterpenes, and geranylgeranyl diphosphate (GGPP), the precursor of diterpenes, carotenoids, side chains of chlorophylls, some vitamins and plastoquinone. The MVA pathway converts acetyl-CoA to IPP and DMAPP to generate farnesyl diphosphate (FPP) in the cytosol, as a precursor mainly of sesquiterpenes and triterpenes. Terpene synthases can cyclize these diphosphated precursors to produce terpene olefins, which are either directly evolved in plant defense or further structured modified to yield complex terpenoids like terpenoid phytoalexins [22,23,24].

Wheat is the main crop plant and cultivated worldwide. Its predominant biotic stresses are catastrophic rust and scab that result in drastic yield reduction and quality deterioration. Disease resistance in wheat has been investigated comprehensively, particularly as to rust resistance [25,26,27,28]. Herbivory is another important factor to diminish wheat yield [29]. For chemical defense against herbivory in wheat, only benzoxazinoids were demonstrated to act as the effective anti-herbivory metabolites [30,31]. Although a few wheat diterpene synthases have been identified to produce different diterpenes [32,33], their final products with modification and biological functions have not been elucidated. Recently, a series of wheat FPP synthases (FPSs) were identified and provided the FPP precursor for increased sesquiterpene production in Arabidopsis, which led to some resistance to aphid infestation [34]. Transient gene silencing of these FPSs in wheat impaired the defense against aphid attack, suggesting that wheat potentially relies on sesquiterpenes in chemical defense, which remains to be clarified [34]. Here, we identified a wheat sesquiterpene synthase that produced β-patchoulene and exhibited to be involved in chemical defense against herbivory.

## 2. Materials and Methods

### 2.1. Plant and Fungus

Wheat (*Triticum aestivum* L. cv Chinese Spring) seeds (provided by Triticeae Research Institute, Sichuan Agricultural University) were germinated and grown in soil at 22 °C with 14 h light/10 h dark for three weeks, ready for treatment. The seeds of *Arabidopsis thaliana* (Col-0) were grown in soil under the condition of 22 °C for 14 h light and 20 °C for 10 h dark period after chilled at 4 °C for three days. *Fusarium graminearum* S. was grown in PDA medium at 28 °C for five days. Spores were collected with sterile water for further inoculation.

### 2.2. Gene Cloning

Three-week-old wheat seedlings were used for RNA extraction with TRIzol reagent (Invitrogen). cDNA was synthesized with M-MLV reverse transcriptase from Takara following the manufactory’s instruction. The *TaPS* gene sequence (Traes_7DL_D9AF9D18B) was acquired from Phytozome for primer design. The coding sequence of *TaPS* was amplified with high fidelity polymerase PrimeSTAR HS (Takara) and ligated into pGM-T vector for sequencing confirmation. For recombinant expression, *TaPS* was subcloned into pET28a with restriction enzymes *BamH* I and *Xho* I.

### 2.3. Recombinant Expression

pET28/*TaPS* was transformed into *E. coli* competent cell line C41(DE3) (Lucigen) for recombinant expression. To provide the isoprenoid precursors, pACYC/*IspA* harboring the *E. coli* FPS gene *IspA* was co-transformed with pET28/*TaPS* following the metabolic engineering approach as described previously [24]. To test the potential monoterpene or diterpene synthase activity of TaPS, genes of IspA S80F (GPP synthase) or GGPP synthase, was also co-transformed with pET28/*TaPS*, respectively [35,36]. Transformed *E. coli* colonies were picked and grown in 5 mL LB medium as the start culture at 37 °C with 200 rpm overnight. The start culture was subsequently transferred into 50 mL LB medium and grown at 37 °C with 200 rpm until reaching 0.8–1 of OD_600_. One mM IPTG was added to induce protein expression and the culture was kept at 16 °C with 200 rpm for continuing growth overnight. Equal volume of hexane was added into the culture to extract terpene products twice. The organic extract was combined and concentrated by rotary evaporation and ready for GC-MS analysis.

### 2.4. GC-MS Analysis

GC-MS analysis was performed on a Shimadzu GCMS-TQ8040 instrument with quadrupole mass spectrometer at EI mode and a DB5 GC column. One μL sample was injected in the splitless mode and analyzed with the following program: 70 °C for 2 min, raising to 250 °C with the rate of 10 °C/min, and kept at 250 °C for 2 min. Compound identification was carried out with mass spectrum comparison using the NIST MS library and the concordance of the retention index.

### 2.5. Gene Expression Analysis and Terpene Detection in Wheat

To analyze *TaPS* gene expression in response to different elicitations, three-week-old wheat seedlings were used for treatment. For pathogen infection, wheat leaves were cut with a scalpel and inoculated with *F. graminearum* spores (10^6^ mL^−1^ in 0.1% Tween 20) as described previously [37], and kept at 100% humidity for 48 h, ready for sample collection. UV treatment was conducted with 254 nm UV light to irradiate wheat seedlings with 15 cm distance for 30 min, and treated samples were kept in the dark for 24 h and transferred into the growth chamber with normal growth condition for 48 h, ready for collection. Methyl jasmonate (MeJA) treatment was performed by spraying 50 μM MeJA on wounded wheat leaves, which were kept at 100% humidity for 48 h and used for RNA extraction. Furthermore, 5 mg/L alamethicin was also used to treat detached wheat leaves for 30 h, which were harvested for later analysis. Wheat leaves were also fed by beet armyworm larvae (third instar) for 30 h and adjacent tissues (~1 cm) were collected for *TaPS* gene expression analysis. Untreated tissues including leaves, stems and roots were also collected for gene expression analysis. All samples were collected for RNA extraction and gene expression analysis by qRT-PCR. The qRT-PCR analysis was performed on ABI StepOne Plus Real-Time PCR System (Thermo Fisher Scientific) with SYBR green mix (Gangchi Bio). Wheat *Actin* was used as the endogenous control gene. All primers used are listed in Table S1 with confirmation by amplicon sequencing. Leaves treated by alamethicin were also used for terpene extraction. Five g leaves were ground in liquid N_2_ to fine powder and extracted twice with 100 mL hexane. The organic extract was combined and concentrated via rotary evaporation for the GC-MS analysis. β-caryophyllene (10 ng) was added into each sample as the internal standard for β-patchoulene quantification.

### 2.6. Overexpression of TaPS in Arabidopsis

To overexpress *TaPS* in plants, *TaPS* was subcloned into pCAMBIA3301 under control of the 35S promoter and subsequently transformed into *Agrobacterium tumefaciens* GV3101. *A. thaliana* Col-0 was used for Agrobacterium transformation through flower dipping. Transgenic plants were screened with spraying of 100 mg L^−1^ glufosinate until T3 generation. Overexpression plants were obtained and two independent lines were randomly chosen for further analysis. The positive T3 plants were analyzed by RT-PCR for *TaPS* gene expression. The amplified fragments were verified by sequencing. The above-ground tissues of T3 plants were ground as fine powders in liquid N_2_ for terpene extraction with hexane as described above, ready for GC-MS analysis. β-patchoulene quantification was conducted as above.

### 2.7. Anti-Herbivory Assays

Arabidopsis plants overexpressing *TaPS* were used for anti-herbivory assays with beet armyworm larvae at the third-instar stage. Two overexpression lines were used for assays independently. For the dual choice assay, seven pieces of transgenic Arabidopsis leaves were collected and placed into one side of the plate with the moist filter paper, and the same numbers of WT leaves were placed into the opposite side of the same plate. Five larvae were starved overnight and placed on the middle line of the plate. The feeding processes were monitored and recorded with photographing. The photos of leaves before and after feeding were analyzed with the Image J software to calculate the consumed leaf area. For the growth inhibition assay, transgenic and WT leaves were placed into separate plates and same numbers of larvae were added into these plates for feeding. Larvae and remained leaves were weighed at 24 h post feeding to determine larvae growth and relative weight gain of larvae. Long-term toxicity was also tested with neonate larvae feeding for 10 d. All experiments were performed with at least three biological replicates.

### 2.8. Homology Modeling and Site-Directed Mutagenesis

Sequence alignment was carried out with CLC Sequence Viewer 7. The sesquiterpene synthases (sTPSs) were used including PatTps177 (*Pogostemon cablin*, Patchoulol synthase, Q49SP3.1), Nt5EAS (*Nicotiana tabacum*, 5-epi-aristolochene synthase, 5EAS_A), AaADS (*Artemisia annua*, amorpha-4,11-diene synthase, Q9AR04.2), AaGAS (*Artemisia annua*, germacrene A, ABE03980.1), CsTPS1 (*Citrus sinensis*, valencene synthase, AAQ04608.1), GaXC14 (*Gossypium arboretum*, (+)-δ-cadinene synthase, Q39760.1), GhTPS1 (*Gossypium hirsutum*, (−)-germacrene D synthase, NP_001314061.1), PatTpsA (*Pogostemon cablin*, γ-curcumene synthase, Q49SP7.1), SlGCS (*Solanum lycopersicum*, germacrene C synthase, AAC39432.1), ScGAS (*Solidago canadensis*, germacrene A synthase, CAC36896.1), StVS(*Solanum tuberosum*, vetispiradiene synthase, NP_001275171.1), VvVAS (*Vitis vinifera*, Valencene synthase, Q6Q3H2.1), ZmTPS10 (*Zea mays*, (E)-β-farnesene synthase, NP_001105850.2), ZSS1 (*Zingiber zerumbet*, α-humulene synthase, B1B1U3.1).

A three-dimensional model of TaPS was acquired on the SWISS-MODEL server (www.swissmodel.expasy.org) using Nt5EAS (PDB ID: 5EAS) as the template. The 3D structure of β-patchoulene was generated by Chem3D. The docking of β-patchoulene-TaPS was generated using AutoDock (autodock.scripps.edu) [38]. The docking results were visualized with PyMOL.

Site-directed mutagenesis was carried out using the Stratagene QuikChange kit following the manufacturer’s instructions. The mutants were confirmed by sequencing and the confirmed constructs were directly used for recombinant expression as described above.

### 2.9. Statistic Analysis

All data were collected and analyzed to determine significant difference through one way ANOVA analysis with Tukey HSD test.

## 3. Results

### 3.1. Identification of TaPS as a Sesquiterpene Synthase

**Figure 1:**
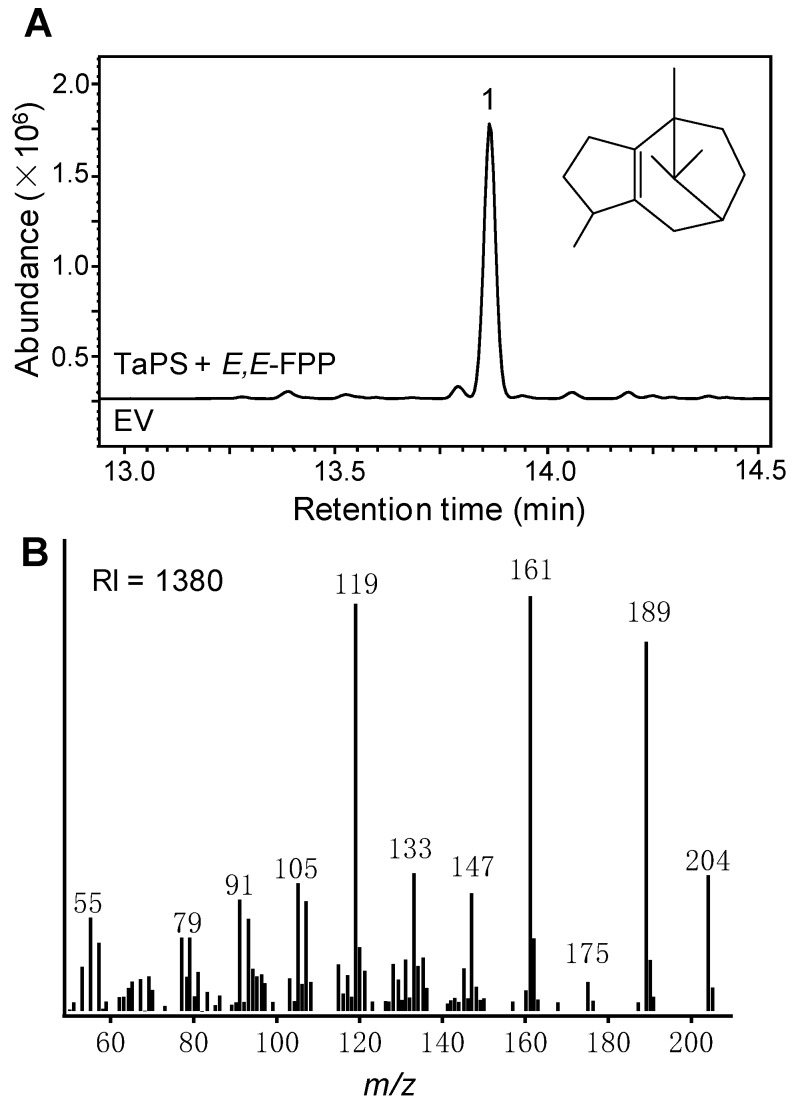
Wheat β-patchoulene synthase (TaPS) catalyzed β-patchoulene formation. (**A**) Gas chromatography-mass spectrometry (GC-MS) chromatogram of extract from the culture co-overexpressing TaPS and farnesyl diphosphate (FPP) synthase in *E. coli*. The empty vector (EV) control was also shown. The product β-patchoulene was labeled as peak 1 and the chemical structure was shown as well. (**B**) Mass spectrum of peak 1. The calculated retention index (RI) was shown.

Terpenes play important roles in plant growth and environmental adaptation. Although a few diterpene synthases have been characterized in wheat, no sesquiterpene synthase was identified. Based on the wheat genome sequence released on Phytozome, we cloned one terpene synthase gene. Transit and/or signal peptide predication indicated no transit/signal peptide sequence in its encoded protein. Recombinant expression in *E. coli* through metabolic engineering enabled to supply isoprenoid precursors for potential terpene synthase [35,39]. This wheat terpene synthase successfully catalyzed a single sesquiterpene production while FPP was supplied through co-expression of an *E. coli* FPP synthase (Figure 1). This sesquiterpene was tentatively identified to be β-patchoulene by the retention index and mass spectrum comparison with that in NIST MS library. GGPP and GPP were also provided to test the potential diterpene or monoterpene synthase activity, respectively; however, this terpene synthase did not exhibit any capacity to catalyze monoterpene or diterpene formation. In combining all these evidences, this wheat terpene synthase gene encoded a β-patchoulene synthase, accordingly named as TaPS.

### 3.2. Inducible TaPS Gene Expression and β-Patchoulene Accumulation

**Figure 2:**
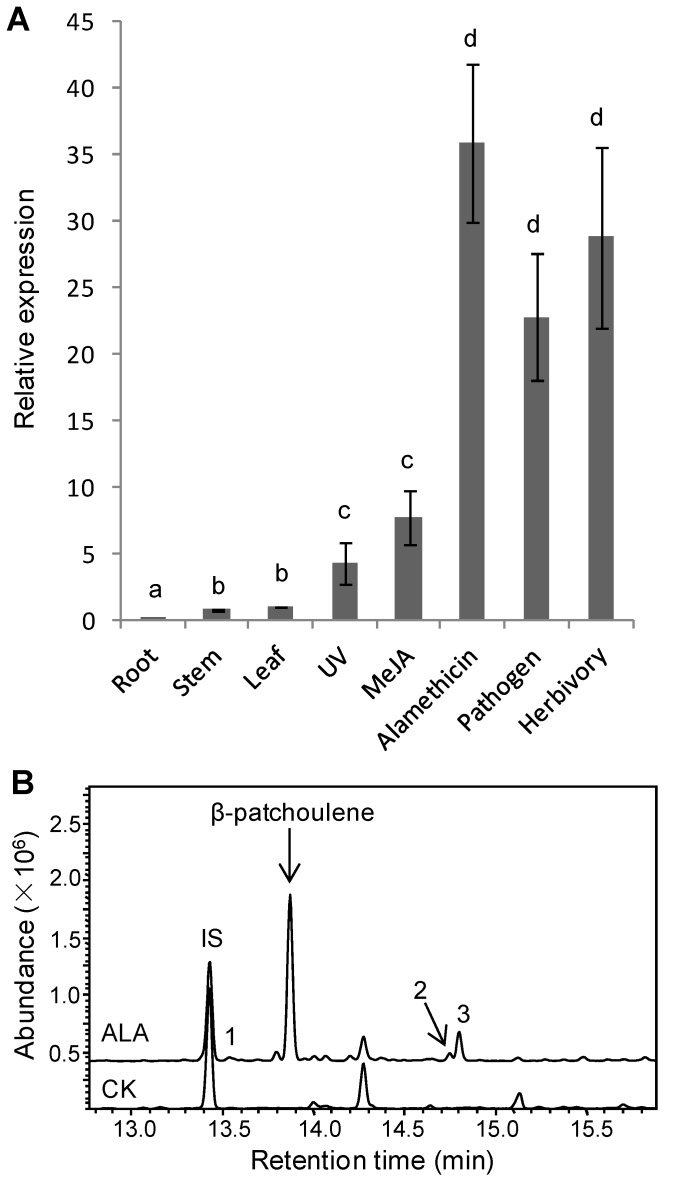
Inducible gene expression of *TaPS* and β-patchoulene accumulation in wheat. (**A**) qRT-PCR analysis of *TaPS* in wheat treated with various elicitations including UV irradiation, 50 μM methyl jasmonate (MeJA), 5 mg/L alamethicin, pathogen infection with *F. graminearum* spore inoculation or herbivory with beet armyworm larvae feeding. Untreated wheat leaves were used as the control. Relative gene expression level of TaPS in roots and stems were also shown. Gene expression of *TaPS* was normalized with wheat *Actin*, the endogenous control gene. Error bars indicate SE (*n* = 3). Different lowercase letters indicate significant difference (Tukey HSD test, *p* < 0.05). (**B**) GC-MS chromatograms of wheat leaf extracts with alamethicin treatment (ALA). CK is the control wheat leaves without any treatments. The induced β-patchoulene and three other sesquiterpenes (peak 1–3) were labeled. Mass spectra and putative identification of peak 1–3 were shown in Figure S2. IS indicates the internal standard.

To explore the biological function of TaPS, its gene expression pattern was analyzed. As indicated in Figure 2A, *TaPS* exhibited low constitutive expression in wheat leaves, stems and roots. Upon elicitation, *TaPS* gene expression was induced differentially in response to various treatments. UV irradiation and MeJA treatment elevated *TaPS* gene expression only slightly. Alamethicin treatment and *F. graminearum* spore inoculation drastically induced *TaPS* gene expression, suggesting involvement of TaPS in biotic stress response. Consistently, herbivory with beet armyworm larvae feeding also strongly induced *TaPS* gene expression in wheat leaves. Based on the gene expression analysis, alamethicin was the powerful elicitor to induce *TaPS* gene expression, which then was used to treat wheat leaves for terpene analysis by GC-MS. β-patchoulene was clearly detected in treated wheat tissues (~24 ng/g FW), this for the first time, but not in controls (Figure 2B), consistent with inducible gene expression of *TaPS*. In addition, we also detected other sesquiterpenes in wheat leaves, suggesting the presence of unknown sesquiterpene synthases that await their identification in future investigation (Figure 2B and Figure S2).

### 3.3. Arabidopsis Overexpressing TaPS Produced β-Patchoulene to Repel S. Exigua Larvae

**Figure 3:**
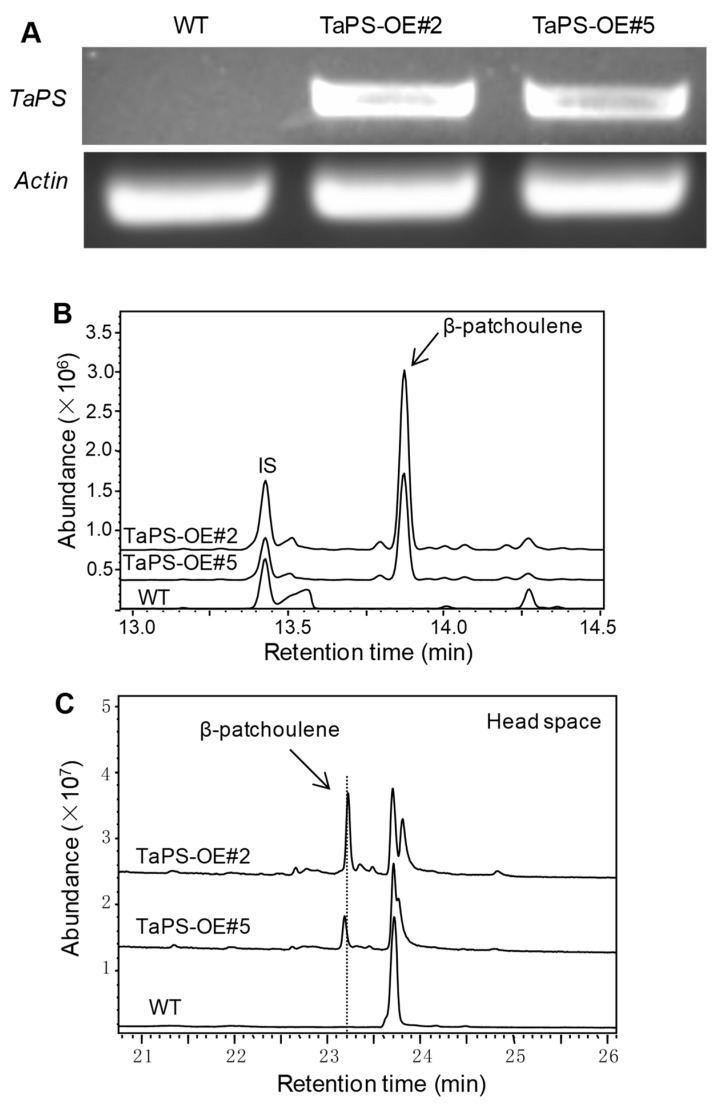
Overexpression of *TaPS* in Arabidopsis. (**A**) RT-PCR analysis of *TaPS* gene expression in transgenic Arabidopsis. *Actin* was used as the control gene; (**B**) GC-MS analysis of β-patchoulene production in two transgenic Arabidopsis lines (TaPS-OE#2 and #5). IS indicates the internal standard; (**C**) head space volatile analysis by GC-MS for *TaPS* transgenic Arabidopsis plants.

To explore the biological function *in planta*, *TaPS* was overexpressed in Arabidopsis under control of the 35S promoter. The RT-PCR analysis indicated that *TaPS* was successfully expressed in Arabidopsis (Figure 3A). Further GC-MS analysis also showed β-patchoulene production (~130 ng/g FW) in two transgenic Arabidopsis lines (Figure 3B,C). These results proved that TaPS catalyzed β-patchoulene formation as a sesquiterpene synthase *in planta*, consistent with its biochemical activity in microbial metabolic engineering system (Figure 1).

**Figure 4:**
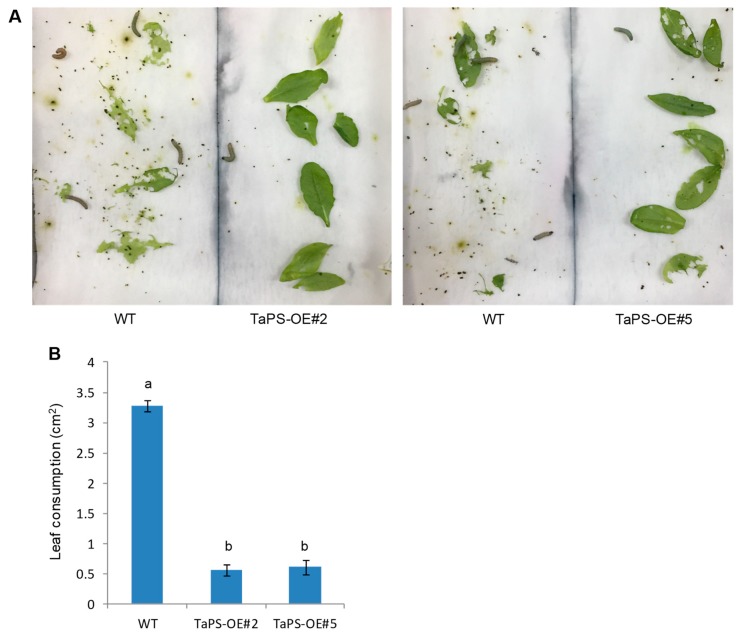
Dual choice assays. (**A**) Arabidopsis leaves fed by beet armyworm larvae for 24 h. Seven pieces of leaves for wild type (WT) or overexpression lines (TaPS-OE) were placed on the opposite side of the same plate. Five larvae were placed at the middle line in each plate. Two independent lines (TaPS-OE#2 and #5) were used for assays; (**B**) leaf consumption of beet armyworm larvae on WT and transgenic Arabidopsis leaves. The consumed leaf area (cm^2^) was calculated based on leaf images before and after feeding with analysis by Image J. The lowercase letters indicate significant difference (Tukey HSD test, *p* < 0.05). Error bars indicate SE (*n* = 3).

Sesquiterpenes have been reported to exhibit various functions particularly anti-herbivory activity in plants, being repellent and/or antifeedant or attracting predators or parasitoids [15,16,17,18,19]. β-patchoulene is the component of essential oil from *P. cablin* and has not been identified to be involved in plant defense. We used transgenic Arabidopsis plants overexpressing *TaPS* to investigate the anti-herbivory activity of β-patchoulene. In the dual-choice feeding preference assay, *S. exigua* larvae preferred WT over transgenic leaves (Figure 4A). After 24 h, leaf consumption by larvae on WT leaves was significantly higher than *TaPS* overexpression lines (Figure 4B), indicating repellent activity of β-patchoulene produced by *TaPS* overexpression in Arabidopsis. The potential toxicity of β-patchoulene on larvae was also explored and no significant growth inhibition was observed when larvae fed transgenic Arabidopsis (Figure S3), suggesting that β-patchoulene might not exert a direct toxicity to *S. exigua* larvae.

### 3.4. Exploring TaPS Catalytic Mechanism by Homology Modeling and Mutagenesis

**Figure 5:**
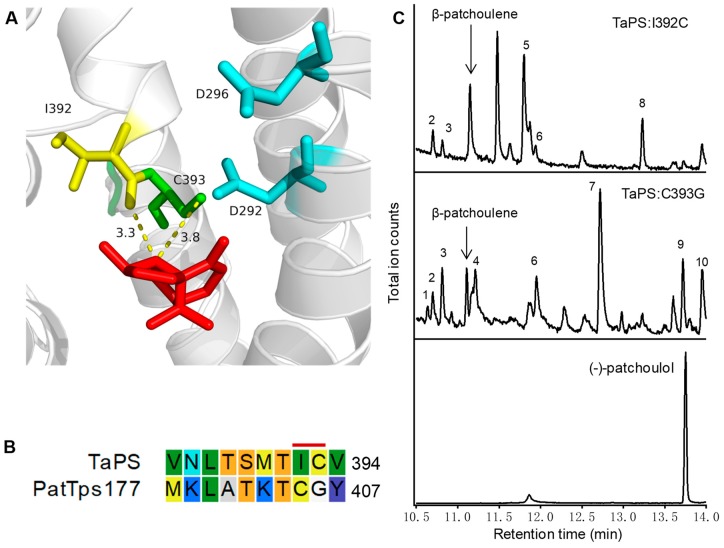
Homology modeling of TaPS and site-directed mutagenesis analysis. (**A**) Catalytic cavity of TaPS was predicted based on homology modeling with docking of the final product β-patchoulene (red). Two key aspartates in the conserved DDXXD motif of terpene synthases were shown in blue. The two unique residues in TaPS, I392 and C393 were shown in yellow and green, respectively. The predicted distances between I392 or C393 and C2 of β-patchoulene were labeled as well (3.3 Å and 3.8 Å, respectively); (**B**) amino acid alignment of TaPS and patchoulol synthase (PatTPS177). The unique I392 and C393 were indicated by the red line. The full alignment was shown in Figure S4; (**C**) GC-MS chromatograms of TaPS:I392C and C393G with FPP as the substrate. The new products were labeled as peak 1–10 and their mass spectra were listed in Figure S5. The authentic (−)-patchoulol was injected for comparison.

To explore the catalytic mechanism of TaPS, homology modeling and docking was performed. As shown in Figure 5A, the residues I392 and C393 were closely localized to the final product β-patchoulene in the kink of Helix G, which was proposed to be involved in stabilization of carbocation in Nt5EAS [40]. Amino acid sequence alignment indicated that both residues of I392 and C393 in TaPS were distinct from equivalent residues of other sTPSs (Figure 5B and Figure S4). Particularly, the patchoulol synthase from *P. cablin* (PatTPS177) has a Cys and Gly at the equivalent positions, respectively [41]. Patchoulol has only one more hydroxyl group than β-pathchoulene and their corresponding biosynthetic enzymes should involve some key residues to generate these two similar products. We mutated I392 and C393 of TaPS to the equivalent C and G of PatTps177, respectively. The product profiles of both mutants were changed with FPP as the substrate (Figure 5C and Figure S5). Notably, some hydroxylated sesquiterpenoids were produced by TaPS mutants. Although none of them was identified as (−)-patchoulol by comparing with the authentic standard, generation of hydroxyl products by these mutants suggests involvement of I392 and C393 in TaPS catalysis and further investigation is needed to clarify the underlying mechanism.

## 4. Discussion

Terpenoids play important roles in plant defense [22]. Terpene volatiles were involved in defense against pests and/or disease pathogens in many plants including maize and rice [42,43,44]. However, terpenoid metabolism in wheat has not been studied thoroughly. Recently, characterization of wheat FPP synthases suggested existence of sesquiterpene biosynthesis in wheat with potential involvement in anti-herbivory response [34]. We searched the wheat genomic sequence released on Phytozome and found a number of genes with annotation of terpene synthase. Here, we identified TaPS from wheat as the β-patchoulene synthase. To the best of our knowledge, TaPS is the first sesquiterpene synthase characterized in wheat.

Alamethicin is usually used to simulate insect herbivory on plants [45]. *TaPS* was induced drastically by the alamethicin treatment in wheat, and it also elicited β-patchoulene accumulation. *TaPS* gene expression and its enzymatic product accumulation responded to alamethicin elicitation, suggesting involvement in the anti-herbivory response. This was confirmed by the inducible gene expression of TaPS in response to beet armyworm larvae feeding on wheat leaves. Furthermore, Arabidopsis overexpressing *TaPS* produced β-patchoulene that acted as the repellent against insect feeding, which also demonstrated *TaPS* involvement in the anti-herbivory response. We further tested the potential toxicity of β-patchoulene to beet armyworm larvae. When the transgenic Arabidopsis leaves were provided as the sole food, larvae did not have other choices and fed on these leaves emitting the disliked β-patchoulene, and slight lower weight gain was observed in comparison with those feeding on WT (Figure S3), indicating no direct toxicity of β-patchoulene on these larvae. In Arabidopsis, semivolatile diterpene, rhizathalene deterred fungus gnat larvae infestation in roots [46]. With artificial diet feeding, fungus gnat larvae consumed less diet containing different concentrations of rhizathalene than the control [46]. In this study, two tested TaPS transgenic lines emitted similar amount of β-patchoulene, which might not reach the toxic concentration. Artificial diet with higher concentration of β-patchoulene should be used for anti-herbivory assays in future studies. Further investigation of such anti-herbivory assays also should be conducted with β-patchoulene spraying on Arabidopsis WT leaves to exclude the potential bad taste of overexpressed TaPS protein in transgenic plants.

A number of volatiles have been identified to defend insect attack through multiple ways in plants. Myrcene, β-caryophyllene and β-farnesene are emitted by plants upon insect damage and acted as the attractors of herbivore enemies [47]. Volatiles also play as the signal molecules to prime defense in neighbor plants [48]. Here we only investigated the antifeedent function of β-patchoulene in Arabidopsis, further study is needed to explore its other potential functions in wheat. Additionally, *TaPS* gene expression and sequence variation should be investigated in wheat varieties and ancestral species to identify its physiological functions and evolution intensively.

Genes encoding enzymes in the biosynthesis of secondary products provide a basis for the production of valuable compounds through metabolic engineering. Patchoulol is an important sesquiterpene responsible for the typical patchouli scent and also used as the intermediate in synthesis of other elaborated products. One patchoulol synthase PatTps177 has been characterized from patchouli, which also produced small amount of patchoulene as the minor product [40]. Here TaPS generated β-patchoulene as the single product and could be used in metabolic engineering for β-patchoulene production specifically. We compared the amino acid sequences of TaPS and PatTps177 and I392 and C393 in TaPS differed from those equivalent residues in this patchoulol synthase. Based on the docking result, both residues were mutated into the corresponding residues Cys and Gly, respectively. However, patchoulol was not detected in both mutants’ products, although some hydroxylated sesquiterpenoids were produced. The equivalent residues in Nt5EAS were T402 and T403 [39]. The dipole of triad (T401-T402-T403) in Nt5EAS was responsible for direction and T403 played a role in carbocation stabilization. As shown in the docking result of β-patchoulene with TaPS (Figure 5A), the 5-carbon-ring of β-patchoulene is close to the kink. Although β-patchoulene is near to C393, C2 rather than C6 is on the positive face, which may result in the abnormal introduction of water and production of other hydryoxyl sesquiterpenoids but not patchoulol (Figure 5C and Figure S5). Furthermore, the substitution of Cys to Gly resulted in loss of the dipolar interaction of the thiol group, which may lead to carbocation instability and improper reaction. Alternatively, the thiol group of C393 might have an interaction with a water molecule. When it was substituted, the water molecule was released and quenched the carbocation to generate hydroxyl sesquiterpenoids. Additionally, the mutation I392C increased the stability of carbocation and changed the cation-shift, thereby produced some new products, which resembled the previous description [39,49]. All these suggest importance of these two amino acids in TaPS catalysis.

## Supplementary Materials

The following are available online at https://www.mdpi.com/2073-4425/10/6/441/s1, Figure S1: Terpene synthesis pathway in plant cells, Figure S2: Mass spectra of wheat sesquiterpenes, Figure S3: Growth inhibition assay, Figure S4: Amino acid alignment of TaPS with other sesquiterpene synthases, Figure S5: Mass spectra of TaPS:I392C and C393G products, Table S1: Primers used in this study.

## References

[1] Neilson E.H., Goodger J.Q., Woodrow I.E., Moller B.L. (2013). Plant chemical defense: At what cost?. Trends Plant Sci..

[2] Aljbory Z., Chen M.S. (2018). Indirect plant defense against insect herbivores: A review. Insect Sci..

[3] Schmelz E.A., Huffaker A., Sims J.W., Christensen S.A., Lu X., Okada K., Peters R.J. (2014). Biosynthesis, elicitation and roles of monocot terpenoid phytoalexins. Plant J..

[4] Cheng A.X., Xiang C.Y., Li J.X., Yang C.Q., Hu W.L., Wang L.J., Lou Y.G., Chen X.Y. (2007). The rice (E)-beta-caryophyllene synthase (OsTPS3) accounts for the major inducible volatile sesquiterpenes. Phytochemistry.

[5] Hasegawa M., Mitsuhara I., Seo S., Imai T., Koga J., Okada K., Yamane H., Ohashi Y. (2010). Phytoalexin accumulation in the interaction between rice and the blast fungus. Mol. Plant Microbe Interact..

[6] Kodama O., Xin Li W., Tamogami S., Akatsuka T. (1992). Oryzalexin S, a novel stemarane-type diterpene rice phytoalexin. Biosci. Biotechnol. Biochem..

[7] Huffaker A., Kaplan F., Vaughan M.M., Dafoe N.J., Ni X., Rocca J.R., Alborn H.T., Teal P.E., Schmelz E.A. (2011). Novel acidic sesquiterpenoids constitute a dominant class of pathogen-induced phytoalexins in maize. Plant Physiol..

[8] Christensen S.A., Huffaker A., Sims J., Hunter C.T., Block A., Vaughan M.M., Willett D., Romero M., Mylroie J.E., Williams W.P. (2018). Fungal and herbivore elicitation of the novel maize sesquiterpenoid, zealexin A4, is attenuated by elevated CO_2_. Planta.

[9] Schmelz E.A., Kaplan F., Huffaker A., Dafoe N.J., Vaughan M.M., Ni X., Rocca J.R., Alborn H.T., Teal P.E. (2011). Identity, regulation, and activity of inducible diterpenoid phytoalexins in maize. Proc. Natl. Acad. Sci. USA.

[10] Mafu S., Ding Y., Murphy K.M., Yaacoobi O., Addison J.B., Wang Q., Shen Z., Briggs S.P., Bohlmann J., Castro-Falcon G. (2018). Discovery, biosynthesis and stress-related accumulation of dolabradiene-derived defenses in maize. Plant Physiol..

[11] Kuc J. (1995). Phytoalexins, stress metabolism, and disease resistance in plants. Annu. Rev. Phytopathol..

[12] Rodriguez A., San Andres V., Cervera M., Redondo A., Alquezar B., Shimada T., Gadea J., Rodrigo M., Zacarias L., Palou L. (2011). The monoterpene limonene in orange peels attracts pests and microorganisms. Plant Signal Behav..

[13] Huang M., Sanchez-Moreiras A.M., Abel C., Sohrabi R., Lee S., Gershenzon J., Tholl D. (2012). The major volatile organic compound emitted from arabidopsis thaliana flowers, the sesquiterpene (E)-beta-caryophyllene, is a defense against a bacterial pathogen. New Phytol..

[14] Chiriboga M.X., Guo H., Campos-Herrera R., Roder G., Imperiali N., Keel C., Maurhofer M., Turlings T.C.J. (2018). Root-colonizing bacteria enhance the levels of (E)-beta-caryophyllene produced by maize roots in response to rootworm feeding. Oecologia.

[15] Kunert G., Reinhold C., Gershenzon J. (2010). Constitutive emission of the aphid alarm pheromone, (E)-beta-farnesene, from plants does not serve as a direct defense against aphids. BMC Ecol..

[16] Kollner T.G., Gershenzon J., Degenhardt J. (2009). Molecular and biochemical evolution of maize terpene synthase 10, an enzyme of indirect defense. Phytochemistry.

[17] Kollner T.G., Held M., Lenk C., Hiltpold I., Turlings T.C., Gershenzon J., Degenhardt J. (2008). A maize (E)-beta-caryophyllene synthase implicated in indirect defense responses against herbivores is not expressed in most american maize varieties. Plant Cell.

[18] Brillada C., Nishihara M., Shimoda T., Garms S., Boland W., Maffei M.E., Arimura G. (2013). Metabolic engineering of the C16 homoterpene TMTT in Lotus japonicus through overexpression of (E,E)-geranyllinalool synthase attracts generalist and specialist predators in different manners. New Phytol..

[19] Sohrabi R., Huh J.H., Badieyan S., Rakotondraibe L.H., Kliebenstein D.J., Sobrado P., Tholl D. (2015). In planta variation of volatile biosynthesis: An alternative biosynthetic route to the formation of the pathogen-induced volatile homoterpene DMNT via triterpene degradation in Arabidopsis roots. Plant Cell.

[20] Oldfield E., Lin F.Y. (2012). Terpene biosynthesis: Modularity rules. Angew. Chem..

[21] Hemmerlin A., Harwood J.L., Fau-Bach T.J., Bach T.J. (2012). A raison d’etre for two distinct pathways in the early steps of plant isoprenoid biosynthesis?. Prog. Lipid Res..

[22] Tholl D. (2006). Terpene synthases and the regulation, diversity and biological roles of terpene metabolism. Curr. Opin. Plant Biol..

[23] Tholl D., Lee S. (2011). Terpene specialized metabolism in Arabidopsis thaliana. Arabidopsis Book.

[24] Mao H., Liu J., Ren F., Peters R.J., Wang Q. (2016). Characterization of CYP71Z18 indicates a role in maize zealexin biosynthesis. Phytochemistry.

[25] Duba A., Goriewa-Duba K., Wachowska U. (2018). A review of the interactions between wheat and wheat pathogens: *Zymoseptoria tritici*, *Fusarium* spp. and *Parastagonospora nodorum*. Int. J. Mol. Sci..

[26] Bahrini I., Ogawa T., Kobayashi F., Kawahigashi H., Handa H. (2011). Overexpression of the pathogen-inducible wheat TAWRKY45 gene confers disease resistance to multiple fungi in transgenic wheat plants. Breed. Sci..

[27] Wang F., Lin R., Feng J., Chen W., Qiu D., Xu S. (2015). TANAC1 acts as a negative regulator of stripe rust resistance in wheat, enhances susceptibility to *Pseudomonas syringae*, and promotes lateral root development in transgenic *Arabidopsis thaliana*. Front. Plant Sci..

[28] Juliana P., Singh R.P., Singh P.K., Poland J.A., Bergstrom G.C., Huerta-Espino J., Bhavani S., Crossa J., Sorrells M.E. (2018). Genome-wide association mapping for resistance to leaf rust, stripe rust and tan spot in wheat reveals potential candidate genes. Theor. Appl. Genet..

[29] Drakulic J., Ajigboye O., Swarup R., Bruce T., Ray R.V. (2016). Aphid infestation increases Fusarium langsethiae and T-2 and HT-2 mycotoxins in wheat. Appl. Environ. Microbiol..

[30] Martyniuk S., Stochmal A., Macias F.A., Marin D., Oleszek W. (2006). Effects of some benzoxazinoids on in vitro growth of Cephalosporium gramineum and other fungi pathogenic to cereals and on Cephalosporium stripe of winter wheat. J. Agric. Food Chem..

[31] Stochmal A., Kus J., Martyniuk S., Oleszek W. (2006). Concentration of benzoxazinoids in roots of field-grown wheat (*Triticum aestivum* L.) varieties. J. Agric. Food Chem..

[32] Wu Y., Zhou K., Toyomasu T., Sugawara C., Oku M., Abe S., Usui M., Mitsuhashi W., Chono M., Chandler P.M. (2012). Functional characterization of wheat copalyl diphosphate synthases sheds light on the early evolution of labdane-related diterpenoid metabolism in the cereals. Phytochemistry.

[33] Zhou K., Xu M., Tiernan M., Xie Q., Toyomasu T., Sugawara C., Oku M., Usui M., Mitsuhashi W., Chono M. (2012). Functional characterization of wheat ent-kaurene(-like) synthases indicates continuing evolution of labdane-related diterpenoid metabolism in the cereals. Phytochemistry.

[34] Zhang Y., Li Z.X., Yu X.D., Fan J., Pickett J.A., Jones H.D., Zhou J.J., Birkett M.A., Caulfield J., Napier J.A. (2015). Molecular characterization of two isoforms of a farnesyl pyrophosphate synthase gene in wheat and their roles in sesquiterpene synthesis and inducible defence against aphid infestation. New Phytol..

[35] Cyr A., Wilderman P.R., Determan M., Peters R.J. (2007). A modular approach for facile biosynthesis of labdane-related diterpenes. J. Am. Chem. Soc..

[36] Reiling K., Yoshikuni Y., Martin V., Newman J., Bohlmann J., Keasling J. (2004). Mono and diterpene production in *Escherichia coli*. Biotechnol. Bioeng..

[37] Fu J., Liu Q., Wang C., Liang J., Liu L., Wang Q. (2018). ZMWRKY79 positively regulates maize phytoalexin biosynthetic gene expression and is involved in stress response. J. Exp. Bot..

[38] Morris G.M., Huey R., Lindstrom W., Sanner M.F., Belew R.K., Goodsell D.S., Olson A.J. (2009). Autodock4 and AutoDockTools4: Automated docking with selective receptor flexibility. J. Comput. Chem..

[39] Morrone D., Lowry L., Determan M.K., Hershey D.M., Xu M., Peters R.J. (2010). Increasing diterpene yield with a modular metabolic engineering system in *E. coli*: Comparison of MEV and MEP isoprenoid precursor pathway engineering. Appl. Microbiol. Biotechnol..

[40] Starks C.M., Back K., Chappell J., Noel J.P. (1997). Structural basis for cyclic terpene biosynthesis by tobacco 5-epi-aristolochene synthase. Science.

[41] Deguerry F., Pastore L., Wu S., Clark A., Chappell J., Schalk M. (2006). The diverse sesquiterpene profile of patchouli, pogostemon cablin, is correlated with a limited number of sesquiterpene synthases. Arch. Biochem. Biophys..

[42] Ding Y., Huffaker A., Kollner T.G., Weckwerth P., Robert C.A.M., Spencer J.L., Lipka A.E., Schmelz E.A. (2017). Selinene volatiles are essential precursors for maize defense promoting fungal pathogen resistance. Plant Physiol..

[43] Schnee C., Kollner T.G., Held M., Turlings T.C., Gershenzon J., Degenhardt J. (2006). The products of a single maize sesquiterpene synthase form a volatile defense signal that attracts natural enemies of maize herbivores. Proc. Natl. Acad. Sci. USA.

[44] Sun Y., Huang X., Ning Y., Jing W., Bruce T.J., Qi F., Xu Q., Wu K., Zhang Y., Guo Y. (2017). TPS46, a Rice Terpene Synthase Conferring Natural Resistance to Bird Cherry-Oat Aphid, *Rhopalosiphum padi* (Linnaeus). Front. Plant Sci..

[45] Herde M., Gartner K., Kollner T.G., Fode B., Boland W., Gershenzon J., Gatz C., Tholl D. (2008). Identification and regulation of TPS04/GES, an Arabidopsis geranyllinalool synthase catalyzing the first step in the formation of the insect-induced volatile C16-homoterpene TMTT. Plant Cell.

[46] Vaughan M.M., Wang Q., Webster F.X., Kiemle D., Tantillo D., Coates R.M., Wray A., Askew W., O’Donnell C., Tokuhisa J.G. (2013). Formation of the unusual semivolatile diterpene rhizathalene by the arabidopsis class I terpene synthase TPS08 in the root stele is involved in defense against belowground herbivory. Plant Cell.

[47] Degenhardt J., Gershenzon J., Baldwin I.T., Kessler A. (2003). Attracting friends to feast on foes: Engineering terpene emission to make crop plants more attractive to herbivore enemies. Curr. Opin. Biotechnol..

[48] Arimura G., Shiojiri K., Karban R. (2010). Acquired immunity to herbivory and allelopathy caused by airborne plant emissions. Phytochemistry.

[49] Zhou K., Peters R.J. (2011). Electrostatic effects on (di)terpene synthase product outcome. Chem. Commun..

