# Supplementary material for: A Wheat β-Patchoulene Synthase Confers Resistance against Herbivory in Transgenic Arabidopsis
Source: Genes (Basel). 2019 Jun 10;10(6):441. doi: 10.3390/genes10060441 (PMC6628343; doi:10.3390/genes10060441)
Supplement: Supplementary file 1 [file genes-10-00441-s001.pdf]

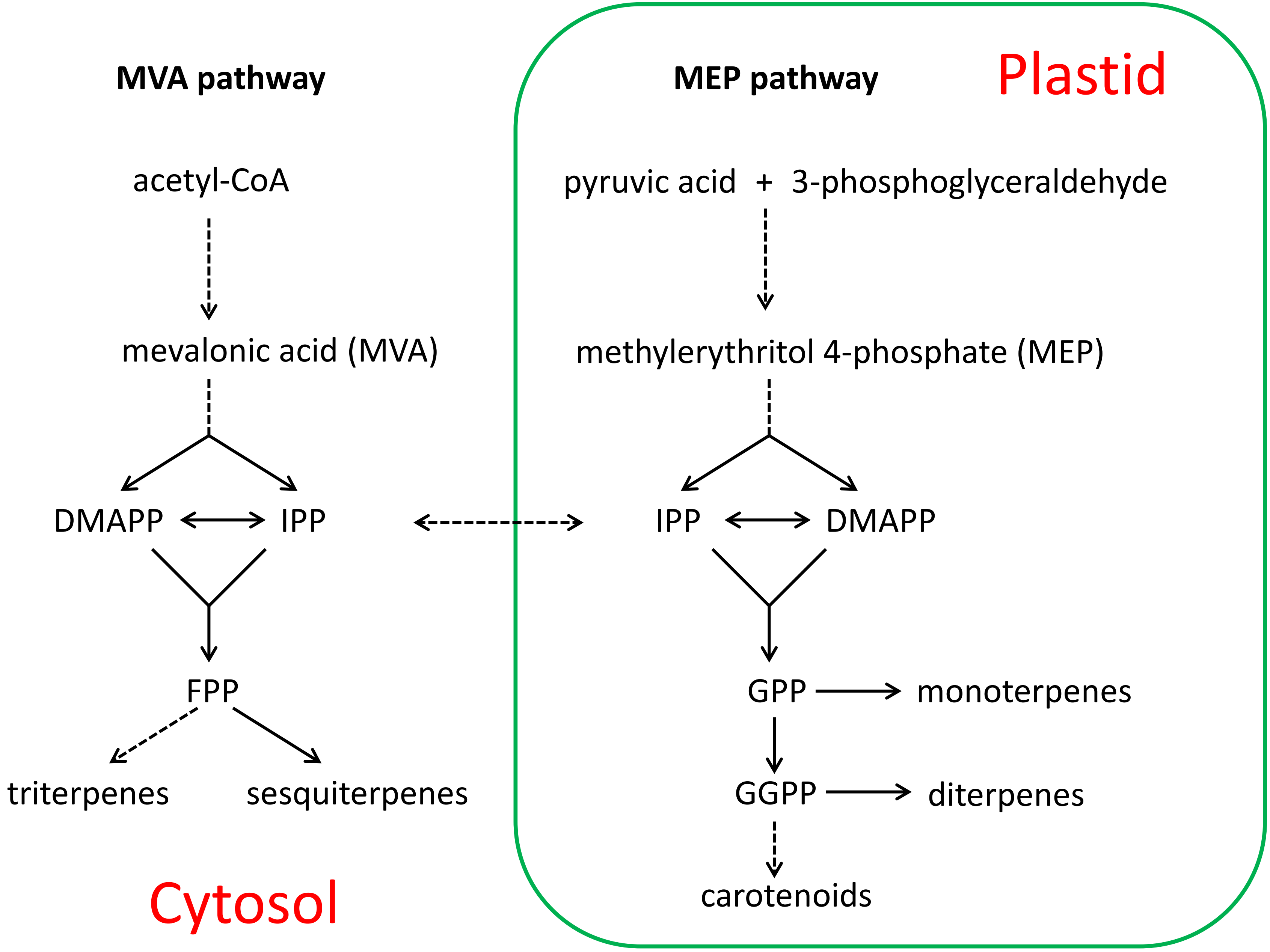

Fig. S1 Terpene synthesis pathway in plant cells

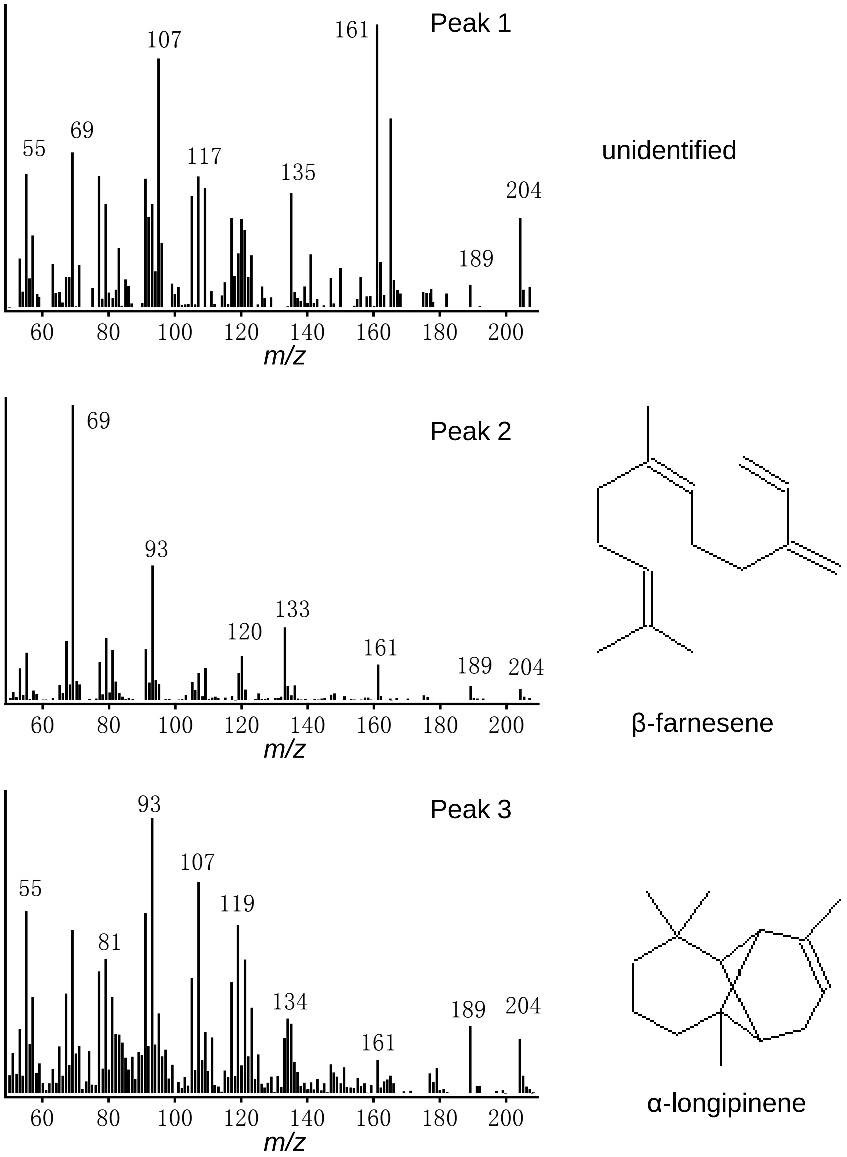

Fig. S2 Mass spectra of wheat sesquiterpenes. Three sesquiterpenes were detected in wheat leaves treated with alamethicin by GC-MS analysis as shown in **Fig. 2B**. Their mass spectra and putative identification were listed here.

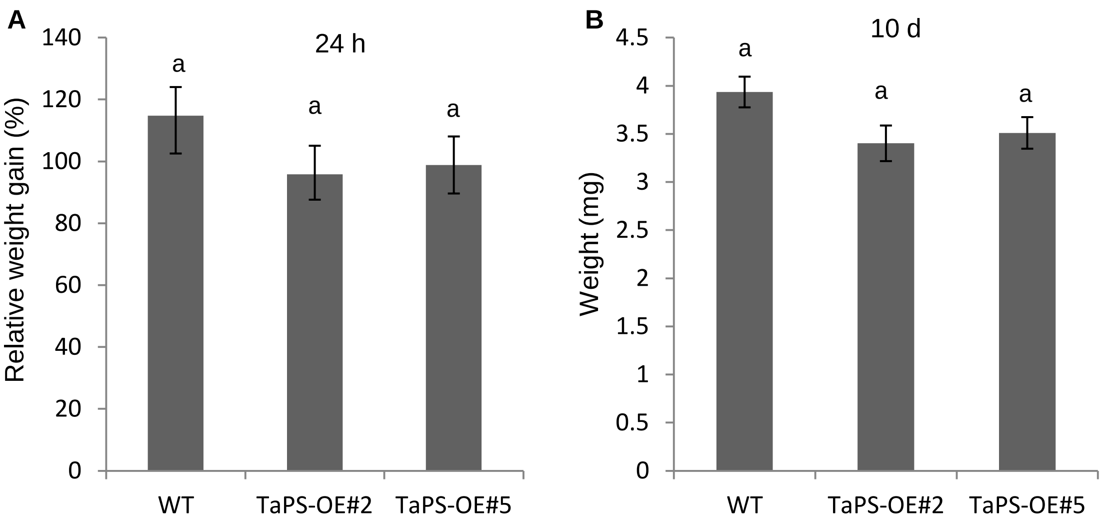

Fig. S3 Growth inhibition assay.
Arabidopsis plants with *TaPS* overexpression were fed by beet armyworm larvae (third instar) for 24 h (**A**) or neonate larvae for 10 d (**B**). Relative weight gain or weight of larvae were determined. WT plants were used as the control. No significant growth inhibition was detected (Tukey HSD test).

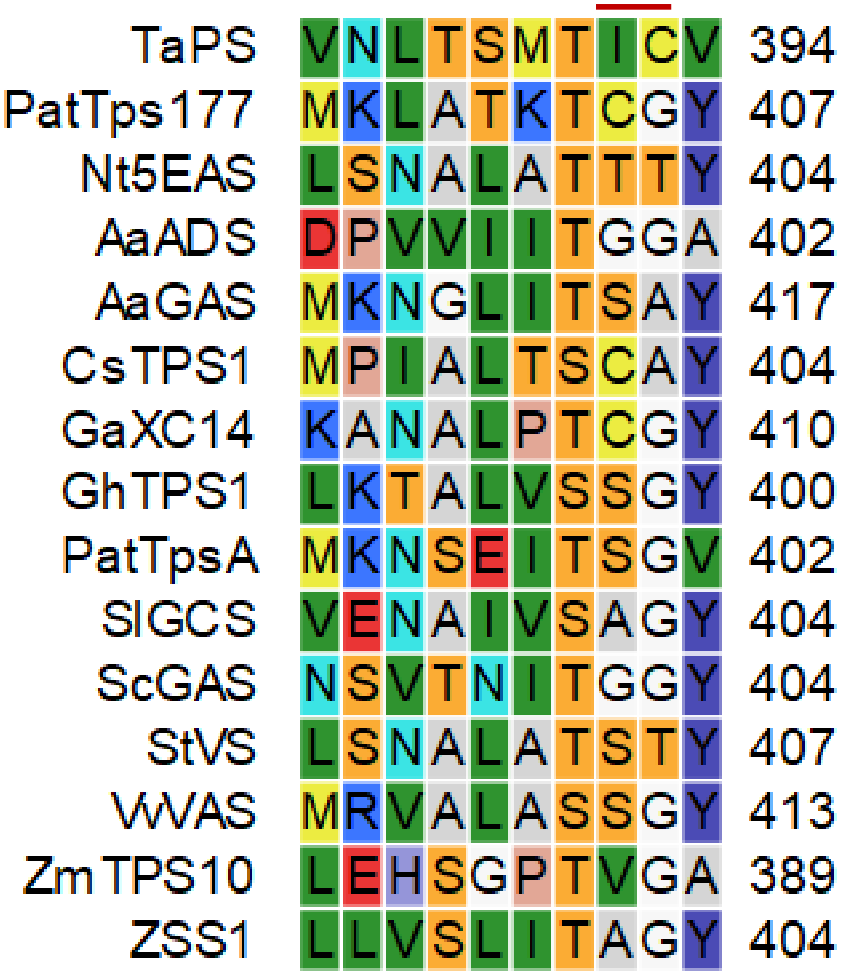

Fig. S4 Amino acid alignment of TaPS with other sesquiterpene synthases.
Equivalent sequences of the kink region in Nt5EAS were shown. The unique I392 and C393 were indicated by the red bold line.

Peak1
RT=10.637min

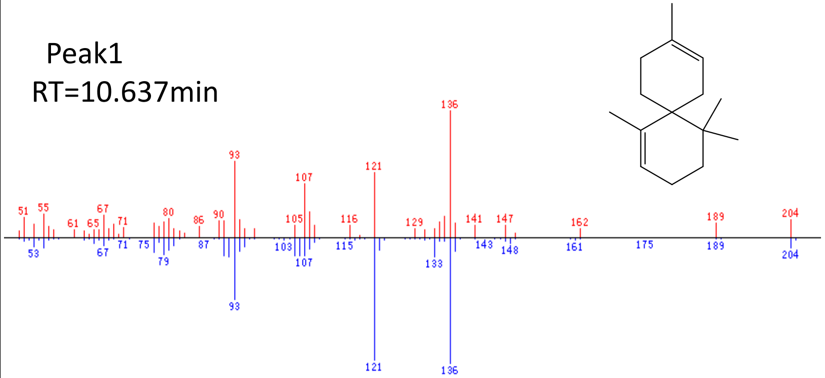

NIST 1,5,5,9-Tetramethylspiro[5.5]undeca-1,9-diene

Peak4
RT=11.124min

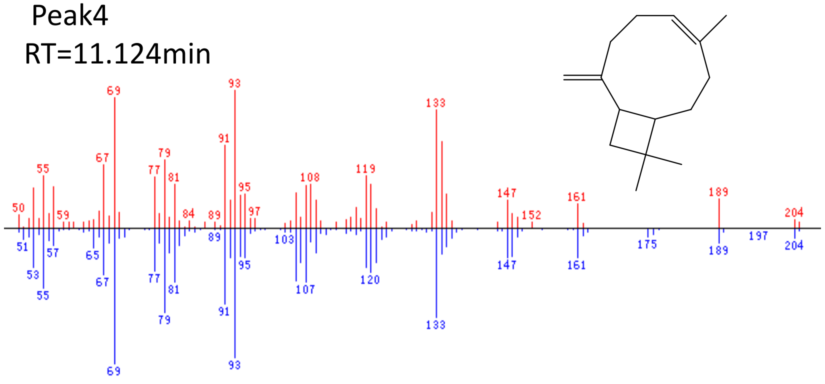

NIST Caryophyllene

Peak 2
RT=10.700min

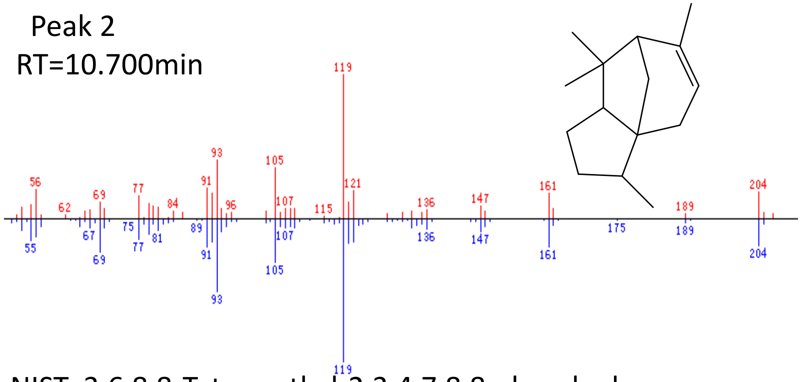

NIST 3,6,8,8-Tetramethyl-2,3,4,7,8,8a-hexahydro-1H-3a,7-methanoazulene

Peak5
RT=11.803min

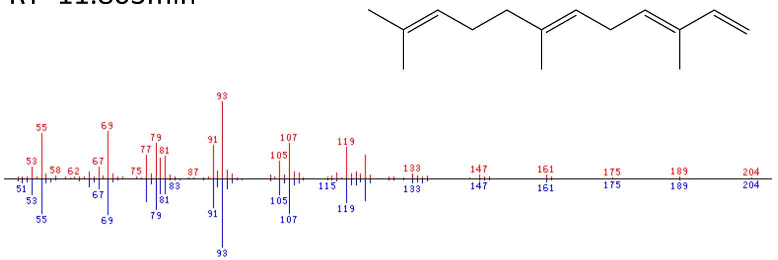

NIST α-Farnesene

Peak3
RT=10.814min

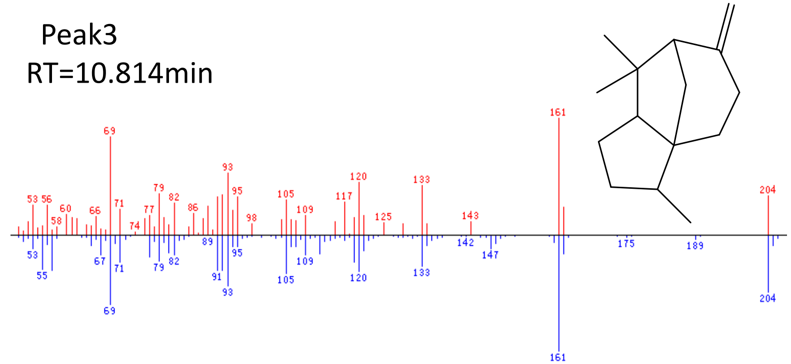

NIST 3,8,8-Trimethyl-6-methyleneoctahydro-1H-3a,7-methanoazulene

Peak 6
RT=11.951min

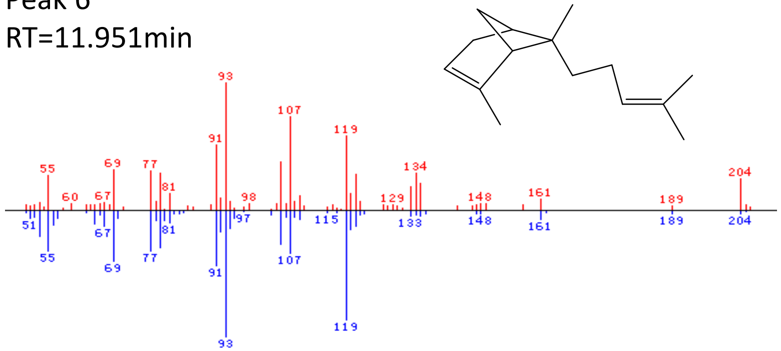

NIST α-Bergamotene

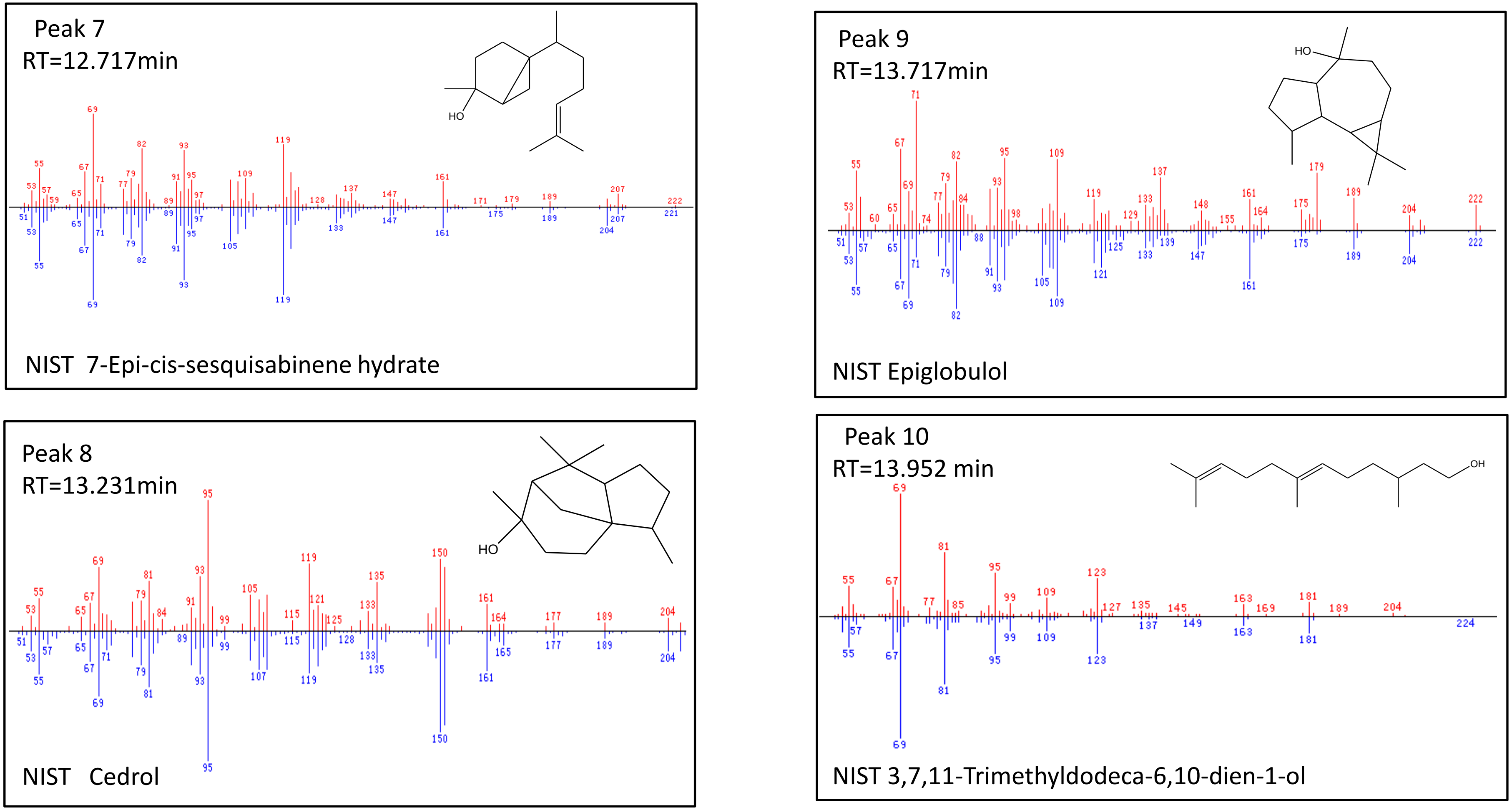

Fig. S5 Mass spectra of TaPS:I392C and C393G products.
Peak 1-10 are products generated by mutants of TaPS:I392C and C393G as shown in Fig. 5c. Their mass spectra are listed here and putative identification were conducted by MS comparison to NIST MS library.

Table S1 Primers used in this study.

**Primer**	**Sequence (5’-3’)**
TaPS-F (qRT-PCR)	GATGTTTCTCTTCTGCCGGAT
TaPS-R (qRT-PCR)	TTTCTTGATGTAGGCAATCTCGT
Wheat Actin-F (qRT-PCR)	AAGTTCCTGGTATACACGAAG
Wheat Actin-R (qRT-PCR)	AGCGGTTGTTGTGAGGGAGT
TaPS-F (RT-PCR)	ATTGTTACTACTTTCAAGATGGCGTCCG
TaPS-R (RT-PCR)	ACCTAGATGGGAATGGGGTTGACGA
Arabidopsis Actin-F (RT-PCR)	GCTGAGCGGGAAATTGTCAG
Arabidopsis Actin-R (RT-PCR)	CCACCGATCCAGACACTGTATTT
